# Cone-Beam Computed Tomography (CBCT)-Based Assessment of the Alveolar Bone Anatomy of the Maxillary and Mandibular Molars: Implication for Immediate Implant Placement

**DOI:** 10.7759/cureus.41608

**Published:** 2023-07-09

**Authors:** Salwa Aldahlawi, Dalia M Nourah, Raneem Y Azab, Jawan A Binyaseen, Ethar A Alsehli, Halema F Zamzami, Omair M Bukhari

**Affiliations:** 1 Dentistry, Umm Al-Qura University, Makkah, SAU

**Keywords:** alveolar bone anatomy, inferior alveolar nerve, maxillary sinus, mandibular molars, maxillary molars, immediate implant, cone beam computed tomography

## Abstract

Purpose

This study aims to examine specific aspects of socket morphology, including buccal and palatal/lingual bone width, interradicular bone (IRB) width, and assessments of root apices and furcation proximity to the vital structures of the maxillary and mandibular first and second molars using cone-beam computed tomography (CBCT).

Materials and methods

The study involved the analysis of 400 maxillary and mandibular first and second molars. Various measurements were taken to assess socket morphology, including mesiodistal (MD) and buccolingual (BL) width, buccal and lingual bone thickness at 2 mm apical to the alveolar crest, IRB width at 2 mm from the furcation, and the distance between the root apices and furcation to vital structures, such as the floor of the maxillary sinus (FMS) and inferior alveolar nerve (IAN).

Results

The mesiobuccal (MB) root of the second molar commonly intruded into the sinus, followed by the palatal root of the maxillary first molar. The mean FMS-F distance was 7.17 + 3.98 mm, and it was 7.2 + 2.72 mm for maxillary first and second molars, respectively. The mean IRB width was 2.77 + 0.96 and 2.29 + 0.74 mm for the first and second molars. The mandibular second molar had the shortest distance to the IAN in comparison to the first molar. For maxillary teeth, 7% of the first and 4% of the second molars presented alveolar anatomy adequate for immediate implant placement, compared to 84% and 50% of mandibular first and second molars.

Conclusion

Understanding the local alveolar bone anatomy of molars and its relationship to vital structures is crucial for the effective planning of implant treatments.

## Introduction

Implant placement immediately after tooth extraction became a standard procedure after its introduction by Bhola in 2008 [1]. It reduces the number of surgical procedures required and decreases the overall treatment time. Immediate implant placement in extraction sockets also limits bone remodeling and reduces the need for bone augmentation [2]. Implants placed in extraction sockets have a comparable survival rate to implants placed in healed sites [3]. The survival rate of 300 immediate implants placed at molar extraction sites was 97.3% after one year [4]. Similarly, a recent systemic review reported a success rate of 93% after one year, with no difference between immediate molar implants placed in the maxilla or the mandible [5].

Primary stability and adequate insertion torque are key factors in the success of immediate implants [6]. The procedure is limited to anterior teeth, as the socket shape allows for the adaptation of the implant to the alveolar wall. Meanwhile, immediate placement at molar sites is more challenging, as the socket anatomy influences whether the primary stability of the implant can be achieved [7]. In addition, anatomical structures, such as the inferior alveolar nerve (IAN) canal or the maxillary sinus (MS), can limit the bone quantity available for implant placement. Various features, including the inter-radical septa dimensions, the distance from the root tips to vital structures, the distance from vital structures to the furcation area, and alveolar bone thickness, affect the bone quantity and quality and are thus critical to the success of the treatment.

Radiography is the most commonly used diagnostic tool in dental offices on a daily basis. Previous studies have supported the use of cone-beam computed tomography (CBCT) for diagnosis, implant treatment planning, anatomical characterization, and treatment outcome evaluation [8]. CBCT is also used to determine the best treatment approach based on individual patient needs.

Previous studies have used CBCT to determine the average distance from the root apices to vital structures in the maxilla [9,10] and mandible [11,12] and between molar furcation and vital structures [13]. However, few studies have addressed other dimensional differences between the first and second molars in the maxilla and mandible. Accordingly, in this study, CBCT data were utilized to assess various measurements related to socked dimensions, including buccopalatal (BL) and mesiodistal (MD) socket width, buccal and palatal/lingual bone width, interradicular bone width (IRB), and assessments of the root apices and furcation proximity to vital structures (e.g., MS in the upper jaw and the IAN in the lower jaw). A comparison of these measurements was performed between the first and second molar sites prior to extraction.

## Materials and methods

Study design

A descriptive retrospective study was conducted using archived CBCT records of dental patients seeking treatment at the University Dental Teaching Hospital. Ethical approval was obtained from the biomedical research ethics committee at Umm Al-Qura University (Approval No: HAPO-02-K-2021-10-809). A convenience sampling technique was used to review patient records from January 2019 to January 2021. CBCT scans of adults over the age of 18 years with at least one molar present were included. Patients who had undergone bone or periodontal regeneration in the posterior area of the maxilla or mandible and who had scans showing evidence of alveolar bone loss, periodontal disease, root resorption, periapical pathosis, or sinus pathosis were excluded.

CBCT images

All CBCT scans were performed using the same machine (i-CAT Vision Q System set at 120 kVp and 37.07 mAs, acquisition time 26.9 sec, and assessed using ICAT Vision viewer, version 1.9.3.13). All images were examined following the same protocol. First, each image was rotated; thus, the long axis was perpendicular to the occlusal plane. The axial view of the central fossa of each molar was then used to determine the sagittal and coronal slices utilized for the measurement. The following measurements were taken.

Maxillary Molars

**Figure 1:**
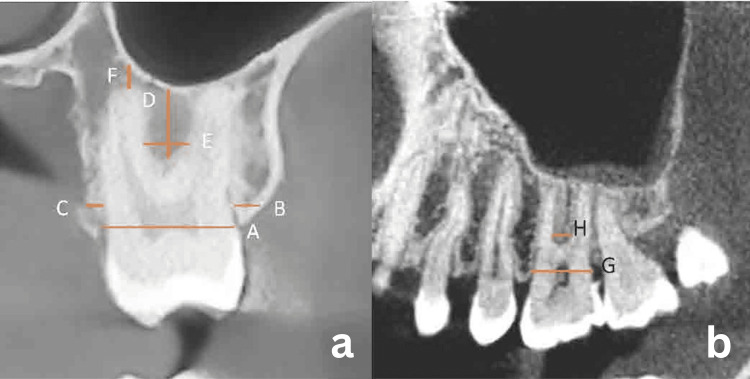
Maxillary molar measurement using CBCT scan a: coronal section. b: sagittal section. A: Buccolingual socket size. B: Width of the buccal bone. C: Width of the palatal bone. D: Distance from FMS to the furcation. E: Width of the interradicular bone 2 mm apical to the furcation. F: Distance from the root apex to FMS. G: Mesiodistal socket size. H: Width of the interradicular bone 2 mm apical to the furcation

Buccal bone thickness (BBT) 2 mm apical to the alveolar crest; palatal bone thickness (PBT) 2 mm apical to the alveolar crest; the distance from the floor of the MS to the furcation (FMS-F); the distance from the floor of the MS to the apex of the palatal root (FMS-P), mesiobuccal root (FMS-MB), and distobuccal root (FMS-DB); the MD and BL socket size at the alveolar crest level; and the width of the interradicular bone 2 mm apical to the furcation (IRB) (Figure 1).

Mandibular Molars

**Figure 2:**
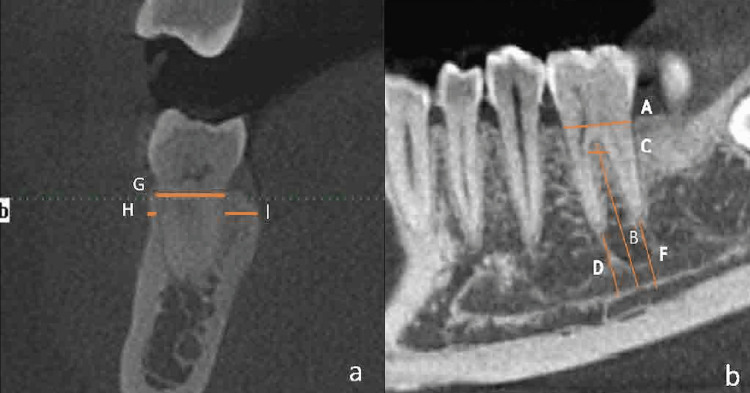
Mandibular molar measurement CBCT scan a: Coronal section. b: Sagittal section. A: Mesiodistal socket size. B: Distance from IAN to the furcation. C: Width of the interradicular bone 2 mm apical to the furcation. D: Distance from the IAN to the apex of the mesial root. F: Distance from the IAN to the apex of the distal root. G: Buccolingual socket size. H: Width of the buccal bone. I: Width of the lingual bone

BBT 2 mm apical to the alveolar crest; lingual bone thickness (LBT) 2 mm apical to the alveolar crest; the distance from the IAN to the furcation (IAN-F); the distance from the IAN to the apex of the mesial root (IAN-M) and distal root (IAN-D); the MD and BL socket size at the alveolar crest level; and the width of the interradicular bone 2 mm apical to the furcation (IRB) (Figure 2).

All examiners were calibrated using a set of 10 CBCT images. Each examiner performed all measurements, and the process was repeated twice, two weeks apart. Since the outcome was continuous, we used Lin’s concordance correlation [14] to quantify inter- and intra-examiner reliability. A sample size of 97 achieved 90% power to detect a difference of 0.5 mm, with an estimated standard deviation of 1.5 and a significance level (alpha) of 0.05 using a two-sided one-sample t-test.

Statistical analysis

Descriptive characteristics are reported as means and standard deviations for continuous variables, numbers, and percentages for categorical variables. Categorical variables were tested using a test of proportions for bivariate analyses, while continuous variables were tested using a paired t-test. To account for correlations within subjects, logistic regression with panel variables such as patient ID and random effects was utilized in the multivariable analysis. This approach enabled the prediction and comparison of distances between different various anatomical landmarks while accounting for correlations within subjects. All statistics were calculated in STATA software (Version 14.2; Stata, College Station, TX). All p-values were two-tailed and interpreted at the 0.05 significance level.

## Results

Four hundred permanent molars (200 mandibular molars and 200 maxillary molars) were evaluated from 121 CBCT images. The study sample included 64 males and 57 females, with a mean age of 32 +13 years.

The inter-examiner reliabilities for the mandibular and maxillary arches were 0.998 and 0.996, respectively, while the intra-examiner reliabilities ranged from 0.996 to 0997, respectively.

Maxillary molars

Alveolar Bone Thickness

The average BBT and PBT of the first and second molars are presented in Table 1. The buccal bone was thicker in the second maxillary molars (2.28 ± 0.88 mm) than in the first maxillary molars (1.58 ± 0.7 mm), with a statistically significant difference of 0.68 mm (95% CI, 0.51 to 0.85, p <0.0001).

**Table 1:** CBCT measurement of the maxillary first and second molars, summarized as mean and standard deviation Buccal bone thickness 2 mm apical to the alveolar crest (BBT); palatal bone thickness 2 mm apical to the alveolar crest (PBT); distance from the floor of the MS to the furcation (FMS-F); distance from the floor of the MS to the apex of the palatal root (FMS-P), mesiobuccal root (FMS-MB), and distobuccal root (FMS-DB); mesiodistal (MD) and buccolingual (BL) socket size at the alveolar crest level; width of the interradicular bone 2 mm apical to the furcation (IRB)

	Maxillary First Molar Mean (+SD)	Maxillary Second Molar Mean (+SD)	P value
BBT	1.58 + 0.7	2.28 + 0.88	0.0001
PBT	1.61 + 0.81	2.15 + 0.98	0.462
FMS-F	7.17 + 3.98 mm	7.2 + 2.72	0.93
IRB	2.77 + 0.96 mm	2.29 + 0.74	0.040
FMS-MB	1.32 + 3.09	-0.46 + 1.72	0.0001
FMS-DB	0.94 + 2.53	0.07 + 1.74	0.002
FMS-P	0.48 + 2.32	0.59 + 1.85	0.348
BL socket size	9.97 + 0.8	7.28 + 0.6	1.55
MD socket size	7.51 + 0.5	9.76 + 0.9	0.002

**Figure 3:**
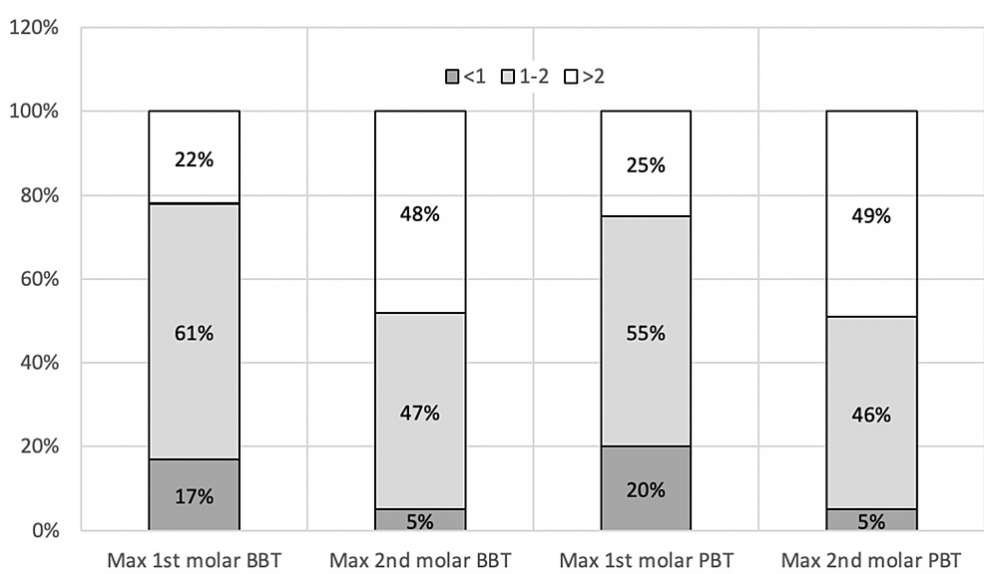
Percentage of maxillary molars with a BBT and PBT of <1 mm, 1-2 mm, and >2 mm BBT: buccal bone thickness; PBT: palatal bone thickness

The percentages of teeth with an alveolar bone thickness <1 mm, 1-2 mm, and >2 mm are presented in Figure 3. Seventeen percent of first molars had a BBT of <1 mm, while 20% of first molars had a PBT of <1 mm. In comparison, only 5% of second molars had a BBT of <1 mm.

FMS-F Distance

**Figure 4:**
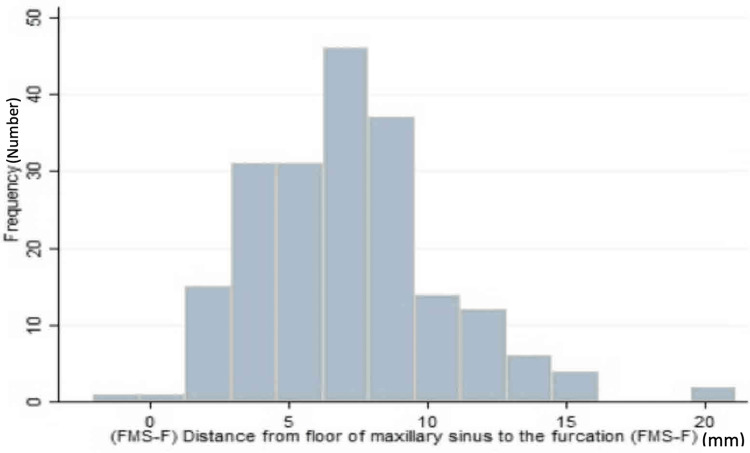
Frequency distribution of the distance from the FMS-F FMS-F: floor of the MS to the furcation

The mean FMS-F distance for maxillary first molars was 7.17 + 3.98 mm, with a range of 1-21.13 mm (Figure 4). The mean FMS-F distance for second molars was 7.2 + 2.72 mm, with a range of 2.06-14.5 mm. The difference was not statistically significant (95% CI, -0.77 to 0.71, p = 0.93) (Table 1). An FMS-F distance of 8 mm is sufficient to place an implant without the need for sinus floor elevation. Only 37% and 35% of the first and second molars, respectively, fulfilled this criterion.

IRB Width

The IRB for the first and second molars was 2.77 + 0.96 and 2.29 + 0.74 mm, respectively (P=0.04 95% CI, -0.77 to 0.71) (Table 1). The odds of having a BBT <1 when the IRB is <3 mm increased by a factor of 5.54 (95% CI, 1.7 to 17.95, p = 0.005).

Root Apex to MS Floor

The mean and standard deviation of the distance between the FMS and MB, DB, and P apexes for the first and second molars are presented in Table 1. The MB root of the second molar had the shortest distance to the sinus, followed by the DB root of the second molar. The MB root of the second molar commonly intruded into the sinus, followed by the palatal root of the maxillary first molar (Table 2).

**Table 2:** Percentage of roots that intrude on, are in contact with, or are 2 mm away from the sinus FMS: floor of the MS; P: palatal; MB: mesiobuccal; DB: distobuccal

Tooth/Distance to FMS	<0	0-1.5 mm	>2 mm
Maxillary First Molar			
P Root	49	31	20
MB Root	25	42	35
DB Root	36	37	27
Maxillary Second Molar			
P Root	30	44	26
MB Root	51	39	8
DB Root	47	32	21

To determine whether there were any significant differences between the roots of first and second maxillary molars in terms of their relationship to the sinus floor, linear regression with the panel variable as patient with random effects was used. On average, the distance from any root to the sinus floor was 0.9 mm longer for the first maxillary molar than for the second maxillary molar (95% CI, 0.60 to 1.20 mm, p <0.0001).

Next, to determine whether there were any significant differences between the roots of the first maxillary molars in terms of their relationship to the FMS, linear regression with panel variable as patient with random effects was used. On average, the distance from the MB root to the sinus floor was 0.85 mm longer in comparison to the palatal root (95% CI, 0.25 to 1.45 mm, p = 0.006). All other comparisons were not statistically significant.

The distance from the DB root of the maxillary second molar to the sinus floor was 0.52 mm shorter compared to the palatal root (95% CI, -0.79 to -0.24 mm, p <0.0001), and the distance from the MB to the sinus floor was 1.05 mm shorter compared to the palatal root (95% CI, -1.32 to -0.77 mm, p <0.0001). In addition, the distance from the MB to the sinus floor was 0.53 mm shorter than the distance from the DB root to the sinus floor (95% CI, -0.8 to -0.25 mm, p <0.0001).

Additionally, when the palatal root was in contact with the sinus, the odds that the mesial or distal roots or both would be in contact with the sinus increased by a factor of 6.34 (95% CI, 1.9 to 21.18, p = 0.003). When the MB was in contact with the sinus, the odds that the DB or P roots or both would be in contact with the sinus increased by a factor of 10.5 (95% CI, 2.88 to 38.31, p <0.0001). Conversely, contact of the DB root with the sinus did not affect the odds of another root having contact with the sinus.

The percentage of first and second molars that exhibited ideal local anatomy for immediate implant placement was determined (i.e., BBT >1 mm, root tip-FMS distance >2 mm, FMS-F distance >8mm, and IRB distance >2 mm). Only 7% of maxillary first molars and 4% of second molars fulfilled all those criteria. Twenty-two percent of maxillary molars required additional procedures, including buccal bone or sinus augmentation, in addition to immediate implant placement.

Mandibular molars

Alveolar Bone Thickness

The average BBT and LBT values of the first and second molars are presented in Table 3. Buccal bone was thicker in second molars (1.74 + 0.45 mm) than in first mandibular molars (1.54 + 0.40 mm), and the difference was statistically significant (p <0.0005).

**Table 3:** CBCT measurement of the mandibular first and second molars, summarized as mean and standard deviation Buccal bone thickness 2 mm apical to the alveolar crest (BBT); lingual bone thickness 2 mm apical to the alveolar crest (LBT); distance from the IAN to the furcation (IAN-F); distance from the IAN to the apex of the mesial root (IAN-M) and distal root (IAN-D); mesiodistal (MD) and buccolingual (BL) socket size at the alveolar crest level; width of the interradicular bone 2 mm apical to the furcation (IRB)

	Mandibular First Molar Mean (+SD)	Mandibular Second Molar Mean (+SD)	P value
BBT	1.54 + 0.40	1.74 + 0.45	0.0005
LBT	1.47 + 0.44	1.73 + 0.42	0.00017
IAN-F	15.89 + 1.98	13.01 + 2.84	0.00001
IRB	2.25 + 0.47	1.81 + 0.45	0.00001
IAN-M	5.31 + 2.35	3.44 + 2.90	0.00001
IAN -D	4.93 + 2.45	2.92 + 2.62	0.00001
BL socket size	8.67 + 1.0	8.74 + 0.7	0.270
MD socket size	8.77 + 1.2	8.87 + 0.6	0.245

**Figure 5:**
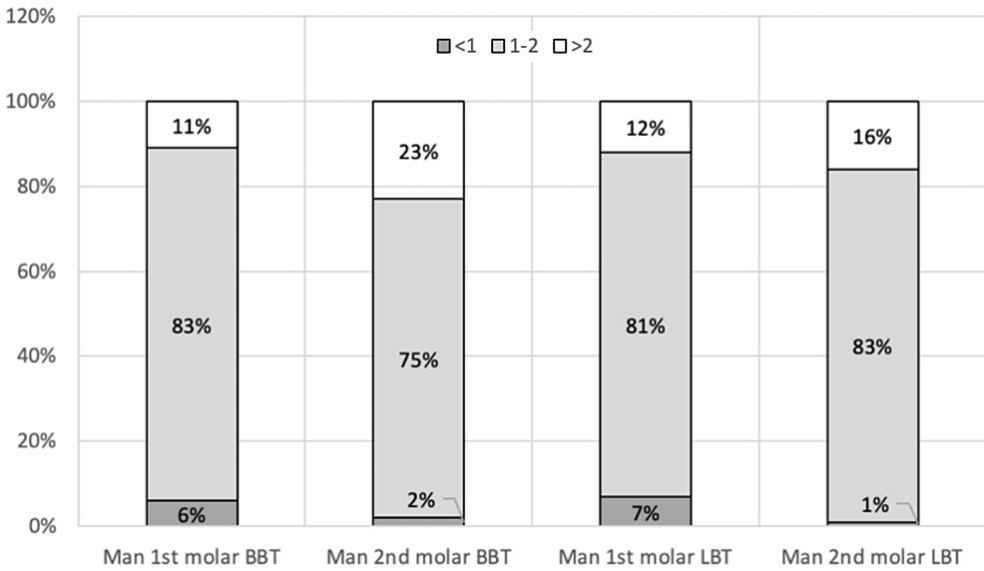
Percentage of mandibular molars with BBT and LBT of <1 mm, 1-2 mm, and >2 mm BBT: buccal bone thickness; LBT: lingual bone thickness

The percentages of molars with an alveolar bone thickness of <1 m, 1-2 mm, and >2 mm are presented in Figure 5. Only 6% of the first molars had a bone thickness of <1 mm, compared to 2% of the second molars (Figure 5).

IAN-F Distance

**Figure 6:**
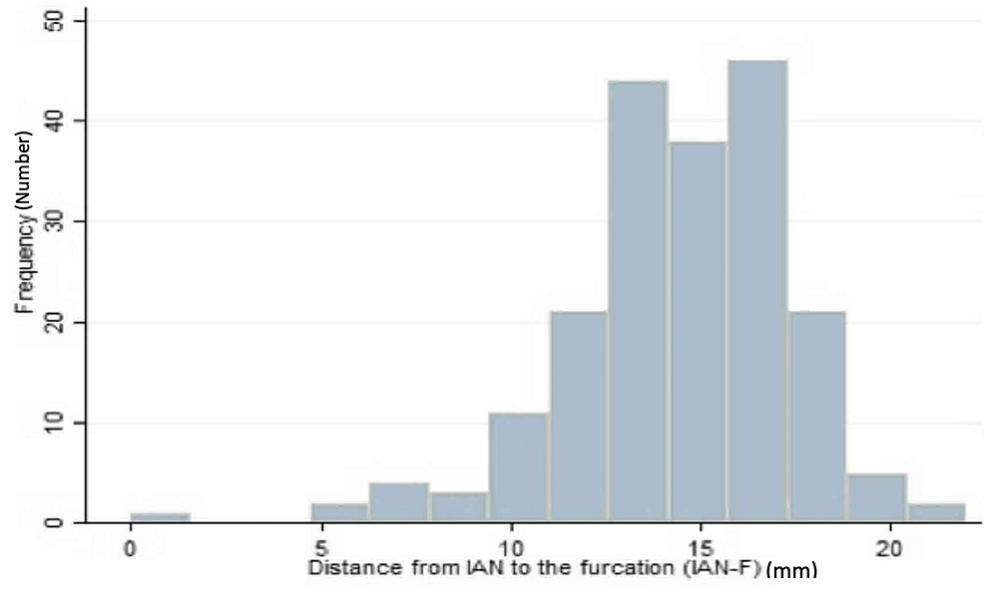
Frequency distribution of the distance from IAN-F IAN-F: IAN to the furcation

The mean IAN-F distance of the mandibular first molars was 15.89 + 1.98 mm, with a range of 10.81 to 22 mm (Figure 6). In comparison, the mean IAN-F distance of the second molars was 13.02 + 2.84 mm, with a range of (5-19 mm). The distance was significantly different (P <0.0001) between the first and second molars (Table 3).

A test was performed to determine whether there was a statistically significant difference between the proportion of first molars and second molars with an IAN-F distance of <10 mm. The proportion of first molars was 11% higher than that of second molars (95% CI, 17.1% to 48.7%, p = 0.0006).

IRB Width

The IRB was 2.25 + 0.47 mm and 1.81 + 0.45 mm for the first and second molars, respectively. A total of 91% of first molars and 100% of second molars had an IRB of <3 mm. A test was conducted to determine whether there was a statistically significant difference between the proportion of first molars and second molars with an IRB of <3 mm. The proportion of first molars was 9% lower than that of the second molars (95% CI, 14.6% to 34%, p = 0.0021).

Root Apex to IAN

The mean and standard deviation of the distances between the IAN and the M and D root apexes for the first and second molars are presented in Table 3. The distance of the roots of second molars to IAN was shorter than that of the roots of first molars (p <0.0001 for both roots), with the distal root of second molars having the shortest distance, followed by the mesial root. The percentages of root apexes in close contact with the IAN are summarized in Table 4.

**Table 4:** Percentage of mandibular molars with roots in contact with/or >2 mm away from the IAN IAN: inferior alveolar nerve; M: mesiobuccal; D: distobuccal

Tooth/Distance to IAN	0-1.5 mm	>2 mm
Mandibular First Molar		
M Root	5	95
D Root	5	95
Mandibular Second Molar		
M Root	31	74
D Root	33	67

When considering teeth with adequate local anatomy for immediate implant placement (i.e., BBT >1 mm, IAN-root tips >2 mm, IAN-F >8 mm, and IRB >2 mm), 84% and 50% of the mandibular first and second molars met those criteria, respectively.

## Discussion

The present study assessed the relationship of maxillary and mandibular molars with vital structures in the upper and lower jaws. It also evaluated local alveolar bone anatomy at molar sites. Inadequate and poor bone quantity influences the insertion torque during immediate implant placement, thereby compromising primary stability and osteointegration [15]. The results showed that the majority of maxillary molars sites don’t have the ideal local anatomy for immediate implant placement and would require a staged approach or additional augmentation procedures for implant rehabilitation. Mandibular molars, on the other hand, are better potential candidates for this treatment approach.

The close relationship between molar roots and the sinus floor has been emphasized in several studies [9,13,16,17]. Some studies have reported that the MB and DB roots of the second molar are those that are most often in contact with the sinus floor [10,18], while others have indicated that the palatal root commonly intrudes into the sinus [19,20]. The differences in these findings could be attributed to differences in the evaluation techniques and study populations and may suggest a racial/ethnic disparity. In this study, the MB of the maxillary second molar most commonly intruded into the sinus, followed by the palatal root of the first molar. Notably, when one root was found to be in contact with or intruded into the sinus, the odds of other roots being in contact with the sinus floor increased by 6.3 and 10.5 for the palatal and MBs, respectively.

The mean FMS-F height in the sample was 7 mm for both maxillary molars, similar to the value reported by Matsuda et al. [9]. This indicates that unless a short implant is used, sinus floor elevation is required to place the implant. The distance between the root apexes and the FMS and the FMS-F distance is essential, as it determines the available residual bone height, influencing the primary stability of immediate implants. It has been reported that at least 2 mm of available height between the root apex and FMS and a minimum 8 mm FMS-F distance are required to place an immediate implant without requiring sinus elevation [4]. Only 37% of the first molars and 35% of the second molars in this study fulfilled these criteria. Similarly, Deporter et al. [13] reported that only 61.7% of maxillary first molars and 34% of second molars are potential candidates for immediate implants.

For replacing maxillary molars, prosthetically driven immediate implant placement involves osteotomy of the furcal bone. A minimum of 3 mm of IRB width is needed for implant placement [12]. Smith and Tarnow [7] classified molar sites into three types according to the availability of furcal bone surrounding the future implant. Most maxillary first molars are type A (IRB >6 mm), while 60% of second molars are type B (IRB = 3-6 mm) [13]. While in the mandible, most extractions sockets were type B in the first molar site and type C in the second molars [21]. In such cases, IRB is insufficient to place the implant with a standard size, and implant primary stability is compromised [22]. In the present study, the mean IRB widths were 2.7 and 2.2 mm for maxillary first and second molars, respectively. However, we only evaluated the IRB distance at the initial root separation (2 mm from the furcation roof). Root separation increases apically, and thus further detailed evaluation of the degree of root separation and furcal bone availability in this population is recommended. Similar to our findings, Beleyan et al. reported that 16% of the molars in their study had initial septal width <3 mm and recommended septal expansions by osseodensification [22]. The osseodensification technique facilitated the preservation of bone and allowed predictable implant placement with adequate stability [22]. When IRB does not support the implant (type C), an ultra-wide implant would be required to engage the socket walls for primary stability. A meta-analysis on the survival of immediate molar implants showed that ultra-wide implants (>6 mm) had a higher failure rate and recommended the use of wide implants (<6 mm) [5].

If the amount of furcal bone is inadequate, the clinician could place the implant at the palatal root site. However, in this study, the FMS-P distance was less than 0.5 mm in both the first and second molars, and the palatal intruded into the sinus in 49% and 30% of the first and second molars, respectively. Other studies have reported similar findings, with palatal root intrusion into the sinus ranging between 29% and 73% and 23% and 69% for first and second molars, respectively [13,18].

Another important consideration when evaluating sites for an immediate implant is the buccal/lingual bone thickness. Thickness plays a significant role in bone fill after implant placement and affects bone resorption during the remodeling phase after extraction [23]. Thinner bone is affected by the interruption of blood supply during the surgical procedure. Bone resorption is also reflected in soft tissue height and stability, and recession and esthetic problems are common complications after immediate implant placement [24]. In this study, most molars had a BBT of 1.5 mm or more [9]. However, some previous studies have reported different results [23]. Mesiodistal and buccopalatal socket measurements are critical for determining the future implant size [25]. Ideally, there should be a 2 mm gap between the implant surface and the buccal bone to ensure the dimensional stability of peri-implant tissue. Filling the jumping gap with a bone graft has been recommended, although it remains a controversial approach [26,27]. Socket measurement is also important in surgical planning, especially if primary closure is required. In this study, the average socket size of maxillary first molars was found to be 10 mm, indicating that a wide-diameter implant can be used.

Regarding mandibular molars, when examining the proximity of the first and second molar roots to the IAN, we found that the second molar roots were significantly closer to the IAN, with the distal root being the closest. A similar finding was reported in several studies [28,29]. We also found that more than one-third of the roots of second molars were in contact with the IAN. Aksoy et al. [28] reported that only 13% of second molar roots and 3% of first molars roots were in contact with the IAN. However, Aljarbou et al. [29] found direct contact between second molars and the IAN for 25% of mesial roots and 38% of distal roots in a Saudi population, with only one case of first molar root contact. The recommended implant height in the posterior molar area is 10 mm [30]. In this study, mandibular first molars often had a greater F-IAN distance than second molars. However, a significantly higher proportion of first molars than second molars had an F-IAN distance of less than 10 mm. This would require either a staged augmentation approach or the use of short implants.

Although a sufficient F-IAN distance was available to place a standard implant, a lack of adequate IRB distance was found in most studied sites. Padhye et al. [12] reported that the mean IRB width in the mandibular first molar was 3.04 mm and that 76% of the evaluated sites lacked adequate bone for implant placement. This prevents complete engagement of the implant with the bone and comprises its primary stability. In this sample, the average BBT was >1.5 mm, with the second molar having significantly thicker buccal bone. Considering all factors, 80% of mandibular first molars and 50% of second molars had a favorable local anatomy for immediate implant placement.

Implant primary stability is one of the predictive factors of achieving osteointegration, especially in immediate implant situations [15,31]. The success of dental implants relies on the integration of the implant with the hard and soft tissue. Implant micromotions during the initial phases of healing have been related to fibrous encapsulation and lack of implant-bone contact [31].

The implant’s primary stability arises from the implant’s mechanical engagement with the surrounding bone, and it depends on many factors including biological factors like bone quantity and bone density [32]. Bone quantity is determined by the available width and height of bone at the future implant site and the proximity to vital structures [33,34]. Studies have shown that 1.5-2 mm of buccal and lingual bone is needed to surround the implants to maintain osseointegration in the long term [35]. Bone augmentation was recommended to achieve adequate width and height in deficient sites [34].

Immediate implants are placed in the extraction socket, and the primary stability of the implants is achieved from the bone apical to the socket, IRB, and from engaging the socket walls. CBCT analysis is essential to find if the local anatomy will provide enough anchorage for the immediate implant by having adequate bone apically without endangering vital structures and laterally by engaging the IRB [8]. In addition, CBCT is also essential in surgical planning by predicting the need for bone augmentation and avoiding complications like MS perforation or IAN injury [8].

Another important factor in predicting implant primary stability is the local bone density at the site of the implant placement. Several studies have shown that bone density measurement on CBCT (voxel grey scales in CBCT and CBCT-based Hounsfield units) correlates with implant stability evaluated at the surgical insertion [36-38] indicating that CBCT is a reliable tool for evaluating bone density preoperatively. Accurate information on bone density will help the operator select suitable implant sites and determine the proper implant design and size to use [37,38]. Modification to the surgical technique such as using osseodensification burs can enhance bone density and improve the insertion torque and, therefore, the primary stability of the implant which reflects the long-term success of the treatment [39].

Limitations of the study

A convenient sampling method was used, which could introduce sampling bias. We evaluated local anatomy based on CBCT and did not include any clinical evaluation. A comprehensive patient evaluation is necessary before determining the best treatment approach. Future research should address other factors that affect the outcomes of immediate implant treatment like local bone density, periodontal phenotype, and the surgical protocol used.

## Conclusions

The data from this study suggest that upper first and second molar sites do not present ideal conditions for immediate implant placement in an accurate position. Most alveolar sites have minimal apical and interradicular bone, which challenges the primary stability of a standard-sized dental implant. Meanwhile, the mandibular first and second molars have the adequate local bone anatomy needed for immediate implant placement.

Accurate preoperative CBCT assessment and knowledge of anatomical measurements, as presented in the current study, are fundamental for planning immediate implants and enable clinicians to identify favorable sites for immediate implant placement as well as sites that may require a staged approach.
